# Genetics in TNF-TNFR pathway: A complex network causing spondyloarthritis and conditioning response to anti-TNFα therapy

**DOI:** 10.1371/journal.pone.0194693

**Published:** 2018-03-26

**Authors:** Ada Aita, Daniela Basso, Roberta Ramonda, Stefania Moz, Mariagrazia Lorenzin, Filippo Navaglia, Carlo-Federico Zambon, Andrea Padoan, Mario Plebani, Leonardo Punzi

**Affiliations:** 1 Laboratory Medicine, Department of Medicine-DIMED, University-Hospital of Padua, Padua, Italy; 2 Rheumatology Unit, Department of Medicine-DIMED, University-Hospital of Padua, Padua, Italy; 3 Department of Biomedical Sciences-BIOMED, University of Padua, Padua, Italy; Taipei Medical University College of Medicine, TAIWAN

## Abstract

**Objectives:**

We investigated whether polymorphisms (SNPs) in the promoter region of *TNFA*, or in the autoinflammatory *TNFRSF1A* and *MEFV* genes, concur with *HLA-B27* in enhancing the risk of Spondyloarthritis (SpA) and/or in predicting the response to anti-TNFα treatment.

**Methods:**

373 controls and 137 SpA (82 with Psoriatic Arthritis-PsA and 55 with Ankylosing Spondylitis- AS; 98/137 under TNFα inhibitor therapy) from the Veneto Region (Italy) were studied. *TNFA* polymorphisms (-1031T>C;-857C>T;-376G>A;-308G>A;-238G>A) and *HLA-B27* were assayed by RT-PCR. Direct sequencing of *MEFV* (exons 2,3,5 and 10) and *TNFRSF1A* (exons 2,3,4 and 6) genes were performed.

**Results:**

*HLA-B27* was associated with AS (χ^2^ = 120.1; p = 0.000). Only the *TNFA* -1031T>C was singly associated with SpA, and the haplotype C/G, resulting from -1031T>C/-308G>A combination, was significantly associated with a reduced risk of SpA (OR: 0.67, CI: 0.46–0.97; p = 0.035). Two SNPs were identified in *TNFRSF1A*, the R92Q (Minor allele frequency-MAF = 0.034) and c.625+10A>G (MAF = 0.479). None of them was associated with SpA (p>0.05). The *TNFRSF1A* c.625+10 G allele was associated with late response to anti-TNFα therapy (p = 0.031). Twenty-one SNPs were identified in *MEFV* gene, 10 with a known potential functional significance. Variant alleles were extremely rare in our population (MAF<0.025) except for R202Q (MAF = 0.27). None was associated with SpA diagnosis (p>0.05).

**Conclusion:**

*TNFRSF1A* and *MEFV* gene SNPs are not associated with SpA in the North-East of Italy. AS risk appears to depend not only on *HLA-B27*, but also on the protective *TNFA* haplotype -1031C/-308G. The *TNFRSF1A* c.625+10A>G impacts on the response to anti-TNFα therapy.

## Introduction

The spondyloarthritis (SpA), a group of chronic inflammatory diseases affecting the sacro ileal joint that includes the two major subtypes Ankylosing Spondylitis (AS) and Psoriatic Arthritis (PsA), results from a complex interplay among genetic background and environmental factors, that leads to the activation of autoinflammation and dys-regulation of the immune-system. Two main groups of SpA have been defined, axial and peripheral SpA, the latter being characterized by the involvement of less than five peripheral joints occurring before, at the same time or after the onset of axial SpA [1,2].

At onset of disease, usually occurring at a young age, SpA diagnosis might be delayed because of the overlap of clinical features and absence of specific blood biomarkers. Although increased serum levels of C-reactive protein (CRP), erythrocyte sedimentation rate (ESR), cytokines, vascular endothelial growth factor (VEGF), calprotectin and matrix metalloproteinase-3 (MMP3) might be observed in SpA patients, these biomarkers have limited specificity and sensitivity, because they are common markers of systemic inflammation and their increase parallels disease activity [3,4]. The delayed SpA diagnosis, estimated to be 8–11 years, underlies a retard in treatment and imparts a tremendous symptomatic burden and loss of function in these patients during the productive years of life [5].

The delay in diagnosis, the lack of specific diagnostic and prognostic biomarkers and of complete clinical response even after therapy with tumor necrosis factor α (TNF-α) inhibitors, such as etanercept, infliximab, adalimumab and golimumab, have raised the interest in the pathogenetic mechanism involved in SpA diseases, with the aim also of identifying biochemical and genetic biomarkers that might help the diagnostic work up and the evaluation of treatment effectiveness [6].

Genes in major histocompatibility complex (MHC) and non-MHC locus have been claimed to play a role in pathogenesis of SpA. The MHC accounts for about 40–50% of the genetic risk of developing SpA, 30% of the total heritability being related to the human leukocyte antigen (*HLA)-B27* haplotype [7]. Single nucleotide polymorphisms (SNPs) in genes involved in TNF-α signalling, as those in the promoter region of *TNFA* gene, in *TNFSF15* (TNF ligand superfamily, member 15), *TNFR-1* (TNF receptor 1) and *TRADD* (TNF receptor type 1-associated death domain protein), have been identified as potentially associated with SpA [8–13]. This is of great interest because TNF-α is not only involved in the propagation and perpetuation of inflammation in SpA, but also because of the clinical efficacy of treatments based on drugs targeting the TNF-α pathway [14,15]. Taking into consideration the potential role of autoinflammation in disease pathogenesis, other potential candidates are the *MEFV* (Mediterranean fever) and *TNFRSF1A* (TNF receptor superfamily member 1A) genes, involved in the pathogenesis of the autoinflammatory disorders Familial Mediterranean Fever (FMF) and Tumor necrosis factor Receptor-Associated Periodic Syndrome (TRAPS) respectively [16,17]. The latter gene encodes the TNFR-1 and genetic variations might underlie functional alterations of the TNF-α signalling, while variations in *MEFV* gene were shown to be very common among FMF Turkish patients with AS as a sole clinical manifestation [18–24].

The aim of the present study, conducted in 137 SpA patients and 373 controls coming from the North-East of Italy, was to verify whether in addition to the established *HLA-B27* genetic predisposing factor, biomarkers of inflammation, SNPs in the promoter region of *TNFA*, or variants of the autoinflammatory *TNFRSF1A* and *MEFV* genes, might be of help in SpA diagnosis and/or in predicting the response to anti-TNFα treatment.

## Patients and methods

### Ethic statement

This case-control study was approved by the Institutional Review Board of the University-Hospital of Padova (Protocol number: 3024P/13) and all subjects gave their fully informed written consent at enrolment.

### Study population

A total of 510 cases and controls, coming from the Veneto Region, a North-East Italian region, were enrolled from January 2014 to December 2017. Cases were 137 SpA patients (84 males, 53 females; mean age ± SD: 51.6 ± 12.6 years) and controls were 373 healthy blood donors (230 males, 143 females; mean age ± SD: 46.5 ± 9.8 years).

Among cases, 55 patients (40%) had a definite diagnosis of AS, according to the modified New York criteria [25] and 82 (60%) had a diagnosis of PsA according to the ClASsification criteria for Psoriatic ARthritis (CASPAR criteria) [26].

All patients underwent clinical, clinimetric and functional examinations following a standardized protocol. Detailed demographic and clinical characteristics of the enrolled patients are shown in Table 1.

**Table 1:** Demographic, clinical characteristics, laboratory indices and outcome measures. **The demographic characteristics were those obtained at enrolment, while patients’ clinical data and laboratory indices are referred to findings obtained at diagnosis of AS or PsA disease**.

	Controls	AS	PsA	Statistics	
	(n = 373)	(n = 55)	(n = 82)		
**Demographic characteristics at enrolment**					
**Gender** (M/F)	230/143	43/12	41/41	χ^2^ = 1.05	**p = 0.004**
**Age,** mean±SD (years)	46.46 ± 9.81	49.76±13.75	52.85±11.66***	F = 12.47	**p<0.0001**
**Weight,** mean±SD (Kg)	77.39 ± 15.55	73.19 ± 10.52	77.44 ± 13.27	F = 1.89	p = 0.1523
**Height,** mean±SD (cm)	172.43 ± 9.95	174.19 ± 6.56	170.63 ± 8.53	F = 2.35	p = 0.0968
**BMI,** mean±SD (Kg/m^2^)	26.18 ± 7.28	24.06 ± 2.74	26.55 ± 4.08	F = 2.79	p = 0.0623
**Clinical characteristics at diagnosis**					
**Family history of SpA**, n (%)		17 (33)	61 (77)	χ^2^ = 25.80	**p < 0.0001**
**Age at diagnosis,** mean±SD (years)		38.59 ±12.95	40.83 ±12.84	t = 0.95	p = 0.3325
**Inflammatory back pain**, n (%)		50 (98)	52 (66)	χ^2^ = 19.03	**p < 0.0001**
**Peripheral arthritis**, n (%)		11 (21)	74 (94)	χ^2^ = 72.37	**p < 0.0001**
**Enthesitis**, n (%)		44 (85)	56 (71)	χ^2^ = 3.27	p = 0.070
**Buttock pain**, n (%)		46 (88)	22 (28)	χ^2^ = 46.15	**p < 0.0001**
**Dactylitis**, n (%)		1 (2)	39 (49)	χ^2^ = 33.27	**p < 0.0001**
**Uveitis**, n (%)		3 (6)	1 (1)	χ^2^ = 2.14	p = 0.143
**Psoriasis**, n (%)		4 (8)	66 (84)	χ^2^ = 72.51	**p < 0.0001**
**IBD**, n (%)		4 (8)	2 (3)	χ^2^ = 1.91	p = 0.167
**Urethritis/Cervicitis/Diarrhoea**, n (%)		5 (10)	18 (23)	χ^2^ = 3.75	p = 0.053
**Laboratory indices and outcome measures at diagnosis**					
**ESR,** mean±SD (mm/hr)		23.98 ± 13.79	29.74 ± 14.42	t = 5.19	p = 0.0244
**CRP,** mean±SD (mg/L)		6.51 ± 5.20	8.00 ± 6.07	t = 2.10	p = 0.1495
**DAS,** mean±SD		3.46 ± 1.23	3.45 ± 0.85	t = 0.00	p = 0.9696
**BASMI,** mean±SD		3.76 ± 1.45	2.17 ± 1.33	t = 41.55	**p <0.0001**
**BASFI,** mean±SD		5.18 ± 2.45	4.27 ± 2.28	t = 4.73	p = 0.0315
**HAQ,** mean±SD		0.83 ± 0.65	0.81 ± 0.56	t = 0.02	p = 0.8924
**BASDAI,** mean±SD		6.20 ± 1.77	5.84 ± 1.91	t = 1.16	p = 0.2839
**ASDAS-PCR,** mean±SD		2.99 ± 0.60	2.92 ± 0.48	t = 0.45	p = 0.5018

Bonferroni’s test for pairwise comparisons:

*** = p<0.0001 with respect to controls. Significant p values are reported in bold face.

At enrolment, 98 patients (71.5%) were under treatment with TNF-α inhibitors (32 Infliximab, 38 Adalimumab, 21 Etanercept, 4 Golimumab, 2 Ustekinumab and 1 Secukinumab) and 16/98 (16.3%) experienced in their clinical history, at least one switch of TNF-α inhibitor before entering the study. Although there is no consensus regarding the criteria to classify the response to TNF-α inhibitors, the European League Against Rheumatism (EULAR) stated that maximal efficacy usually is not seen before 6 months even when improvement is achieved at 3 months, and indicates monitoring every 6–12 months once the treatment target has been attained [27]. Based on these recommendations, we considered 10 months-evaluation an adequate time point to make a final classification of patients as early or late responders, being responders those who reached a Bath Ankylosing Spondylitis Disease Activity Index (BASDAI) score lower than/equal to 4, on a scale from 0 (no activity) to 10 (high disease activity), in agreement with Braun et al [28].

### Haematological and biochemical indices

The complete blood count, ESR, CRP, glucose, uric acid, creatinine, alanine aminotransferase (ALT) and prealbumin were measured by standard laboratory methods.

### Genetic analyses

Genomic DNA was isolated from EDTA-K_2_ peripheral blood (MagNA Pure System, Roche S.p.A., Monza, Italy). *HLA-B27* was determined using a commercially available CE-IVD microarray (EUROArray HLA-B27–Direct, Euroimmun AG, Luebeck, Germany). The genotype discrimination of five *TNFA* gene SNPs (-1031T>C, rs1799964; -857C>T, rs1799724; -376G>A, rs1800750; -308G>A, rs1800629; -238G>A, rs361525) was performed by a TaqMan dual probes allelic discrimination assay using ABI Prism 7900 (Applied Biosystem, Foster City, CA, USA) as previously described by us [29]. Representative instrumental findings are shown in S1 and S2 Figs.

Exons 2, 3, 4 and 6 of the *TNFRSF1A* gene were studied using denaturing high-performance liquid chromatography (DHPLC; Wave 2100 Fragment Analysis, Transgenomic, Omaha, NE, USA) and gene sequencing (ABI PRISM 3130 Genetic Analyzer, Applied Biosystem, CA, USA) of identified heteroduplexes as described by us elsewhere [30]. Polymorphisms of the *MEFV* gene were analyzed by direct sequencing of exons 2, 3, 5 and 10 (Applied Biosystem, CA, USA) as detailed in S1 File and S1 Table. The genotype discrimination of the R202Q SNP (rs224222) in *MEFV* gene (exon 2) was performed by a TaqMan dual probes allelic discrimination assay (Applied Biosystem, Taqman SNP genotyping, catalogue number C_2394721_10).

All genetic analyses were performed in an exploratory cohort of 91 patients and 27 controls. To improve the estimate of significant associations found at interim analysis in the exploratory cohort, the number of examined controls was extended from 27 to 214 for the *HLA-B27* and to 218 for the R202Q SNP in *MEFV* gene, the maximum number of available biomaterial. The analyses of *TNFA* SNPs were extended to all cases and controls for whom biological material was available. The following data were available: -1031 T>C SNP 228/373 controls and 136/137 patients; -857C>T SNP: 368/373 controls and 135/137 patients; -376G>A SNP: 254/373 controls and 130/137 patients; -308G>A SNP: 365/373 controls and 135/137 patients; -238G>A SNP: 356/373 controls and 137/137 patients.

### TNF-α mRNA expression analysis

Peripheral blood mononuclear cells (PBMCs) obtained from 95 controls, 19 AS and 26 PsA were isolated by gradient centrifugation (Histopaque, 1077, Sigma-Aldrich, Milano, Italy). Total RNA from PBMCs was isolated according to the manufacturer’s instructions (High Pure RNA Isolation Kit, Roche, Germany). Five hundred ng of total RNA were reverse transcribed into cDNA (Random primers and Superscript^TM^ II -Reverse Transcriptase, Invitrogen, Italy). Relative quantification of TNF- α was undertaken by RT-PCR with ABI Prism 7900 HT (Applied Biosystems, CA, USA) using the primers and probe set supplied by ThermoFisher Scientific (Waltham, USA, catalogue number 4331182, assay ID Hs00174128_m1). Relative quantifications were performed in a final volume of 20 μL with 33 ng of cDNA. The reference gene, HPRT1 (Hypoxanthine-guanine phosphoribosyltransferase), was selected according to the method commonly used for internal control for quantitative gene expression analyses, and its expression was determined by commercially available HPRT1 primers and probe sets (Part Number 4326321E, Applied Biosystems, CA, USA). TNF and HPRT1 were analyzed in duplicate for each sample. PCR was run at 2 minutes at 50°C, 10 min at 95°C, followed by 40 cycles of 15 seconds at 95°C and 1 min at 60°C. To determine the relative mRNA expression levels of TNF gene we used the comparative Ct method, a mathematical model that calculates changes in gene expression as a relative fold difference between an experimental and a pool derived from 20 healthy blood donors used as calibrator sample.

### Statistical analysis

The chi-square (χ^2^) test, Fisher’s exact test, binary logistic regression analysis, Student’s t test for unpaired data, analysis of variance (ANOVA) and Bonferroni’s adjustment of p value for multiple testing were performed using Stata software, version 13.1 (StataCorp, Lakeway Drive, TX, USA). Genotype frequencies were tested for Hardy-Weinberg equilibrium proportions using χ^2^ test. Haplotypes phases and frequencies were estimated by the retrospective profile-likelihood approach using the Stata haplologit package. Odds ratio (OR) was calculated by logistic regression analysis using the additive model [31].

## Results

### Biomarkers of inflammation in AS and PsA

Based on the premise that inflammation of the sacroiliac joint is the hallmark of SpA, we first verified whether this confined inflammatory process could induce alterations in haematological and biochemical inflammatory indices.

The results found at enrolment in cases and controls are reported in Table 2. Both AS and PsA were associated with haematological and biochemical signs of systemic inflammation, being the number of white blood cells (WBC), polymorphonuclear cells (PMN) and platelets (PLT), and serum CRP levels increased in patients with respect to controls. PsA was also correlated with increased levels of glucose and ALT.

**Table 2:** Haematological and biochemical data in controls, AS and PsA patients at enrolment.

	Controls (n = 373)	AS (n = 55)	PsA (n = 82)	Statistics	
	Mean ± SD	Mean ± SD	Mean ± SD		
**WBC** (x10^3^/μL)	5.60 ± 1.32	7.02 ± 2.16***	7.06 ± 1.93***	F = 22.30	**p<0.0001**
**Haemoglobin** (g/L)	144.60 ± 12.8	144.03 ± 12.68	143.87 ± 13.62	F = 0.09	p = 0.9162
**Platelets** (x10^3^/μL)	238.05 ± 48.67	252.13 ± 67.23	261.37 ± 59.67**	F = 6.78	**p = 0.0013**
**PMN** (x10^3^/μL)	3.23 ± 1.006	4.07 ± 1.70***	4.04 ± 1.51 ***	F = 20.40	**p<0.0001**
**Monocytes** (x10^3^/μL)	0.44 ± 0.30	0.48 ± 0.19	0.86 ± 3.57	F = 2.40	p = 0.0918
**Lymphocytes** (x10^3^/μL)	1.87 ± 1.61	2.14 ± 0.80	2.19 ± 0.74	F = 2.03	p = 0.1322
**Glucose** (mmol/L)	4.76 ± 0.90	5.06 ± 0.89	5.07 ± 0.95*	F = 5.02	**p = 0.007**
**Creatinine** (μmol/L)	77.89 ± 12.92	74.11 ± 14.59	74.5 ± 16.68	F = 3.02	**p = 0.050**
**Uric acid** (mmol/L)	0.29 ± 0.06	0.30 ± 0.08	0.29 ± 0.08	F = 0.29	p = 0.7497
**ALT** (U/L)	22.75 ± 9.14	26.19 ± 14.55	29.32 ± 22.00***	F = 8.63	**p = 0.0002**
**Prealbumin** (mg/L)	286.26 ± 44.28	280.14 ± 48.45	275.95 ± 42.98	F = 1.72	p = 0.1808
**CRP** (mg/L)	3.59 ± 3.67	5.61 ± 5.77**	4.92 ± 4.04*	F = 7.74	**p = 0.0005**

Bonferroni’s test for pairwise comparisons:

* = p<0.05

** = p<0.005

*** = p<0.0001 with respect to controls.

Significant p values are reported in bold face.

### *HLA-B27* is associated with AS

The genetic basis of SpA is complex, but the role of *HLA-B27* haplotype is well established. For this reason we first evaluated in a series of 214/373 controls and 91/137 patients the *HLA-B27* haplotype, results being presented in Table 3. As expected, SpA cases carrying the *HLA-B27* were more frequent than controls (χ^2^ = 120.12, p< 0.0001), being this haplotype mainly associated with AS than with PsA.

**Table 3:** *HLA-B27* haplotype in AS and PsA cases and controls.

Controls (n = 214)		AS (n = 36)			PsA (n = 55)		
**Number**		**Number**		**Statistics#**	**Number**		**Statistics#**
**(Frequency, %)**		**(Frequency, %)**			**(Frequency, %)**		
***Carriers***	***Non carriers***	***Carriers***	***Non carriers***	χ^2^ = 118.41	***Carriers***	***Non carriers***	χ^2^ = 5.68
9	205	26	10	***p<0*.*0001***	7	48	p = *0*.*051*
(4.21)	(95.79)	(72.22)	(27.78)		(12.73)	(87.27)	

#The chi-square tests were performed by comparing AS or PsA with controls. Significant p values are reported in bold face.

### *TNFA -1031C/-308G* haplotype reduces AS risk

A number of variants in genes encoding for inflammatory and immunomodulatory cytokines and for their receptors could be involved in SpA. In this study we investigated the presence of association between SpA and five SNPs in the promoter region of *TNFA*. These SNPs regulate singly or combined in haplotypes the transcription of TNF- α, one of the main cytokines involved in SpA pathogenesis and target of therapy with TNF- α inhibitors.

The genotypes and Minor Allele Frequency (MAF) resulting from the five studied SNPs of the *TNFA* gene and found in patients (AS and PsA) and controls are reported in Table 4.

**Table 4:** *TNFA* gene polymorphisms in controls, AS and PsA patients.

dbSNP	MAF	Controls			AS			PsA			Statistics
*TNFA*		GENOTYPES			GENOTYPES			GENOTYPES			
gene		Number			Number			Number			
		(frequency)			(frequency)			(frequency)			
-1031T>C	C	C/C	T/C	T/T	C/C	T/C	T/T	C/C	T/C	T/T	χ^2^ = 11.97
rs1799964	(0.242)	12	97	119	1	11	42	4	34	44	**p = 0.018**
		(0.05)	(0.43)	(0.52)	(0.02)	(0.20)	(0.78)	(0.05)	(0.41)	(0.54)	
-857C>T	T	T/T	C/T	C/C	T/T	C/T	C/C	T/T	C/T	C/C	χ^2^ = 1.48
rs1799724	(0.217)	18	120	230	4	18	33	3	30	47	p = 0.83
		(0.05)	(0.33)	(0.62)	(0.07)	(0.33)	(0.60)	(0.04)	(0.37)	(0.59)	
-376G>A	A	A/A	G/A	G/G	A/A	G/A	G/G	A/A	G/A	G/G	χ^2^ = 2.69
rs1800750	(0.020)	0	12	242	0	0	55	0	3	72	p = 0.26
		(-)	(0.05)	(0.95)	(-)	(-)	(1)	(-)	(0.04)	(0.96)	
-308G>A	A	A/A	G/A	G/G	A/A	G/A	G/G	A/A	G/A	G/G	χ^2^ = 3.40
rs1800629	(0.109)	5	69	291	0	10	45	0	20	60	p = 0.493
		(0.01)	(0.19)	(0.80)	(-)	(0.18)	(0.82)	(-)	(0.25)	(0.75)	
-238G>A	A	A/A	G/A	G/G	A/A	G/A	G/G	A/A	G/A	G/G	χ^2^ = 8.43
rs361525	(0.055)	0	42	314	0	2	53	1	8	73	p = 0.07
		(-)	(0.12)	(0.88)	(-)	(0.04)	(0.96)	(0.01)	(0.10)	(0.89)	

Significant p values are reported in bold face.

All polymorphisms were in Hardy-Weinberg equilibrium (p >0.01) and only the -1031T>C SNP was significantly correlated with disease diagnosis, with a lower number of AS cases carrying the TC or the CC genotypes.

The studied polymorphisms of the *TNFA* gene, all closely located in the promoter region, are carried as different haplotypes. The haplotypes and their frequencies in controls resulting from the pairwise combination of the five SNPs were inferred by statistical analysis (S2 Table).

To verify whether *TNFA* haplotypes exert any independent role over SpA diagnosis, logistic regression analyses were performed, on considering SpA as the outcome variable and *TNFA* haplotype combinations as predictors. Table 5. reports the OR with 95% confidence intervals (CI) with respect to the reference haplotype (Ref), which, for any combination, was the most frequent in controls.

**Table 5:** Logistic regression analyses considering SpA diagnosis as the outcome variable and *TNFA* haplotype combinations as predictors.

SNP	SNP	*TNFA* haplotypes	OR (95% CI)	p
-1031T>C	-857C>T	-1031T/-857C	Ref	
		-1031C/-857C	0.69 (0.47–1.05)	0.071
		-1031T/-857T	0.99 (0.69–1.40)	0.946
		-1031C/-857T	-	-
-1031T>C	-376G>A	-1031T/-376G	Ref	
		-1031C/-376G	0.71 (0.49–1.03)	0.072
		-1031C/-376A	-	-
		-1031T/-376A	-	-
-1031T>C	-308G>A	-1031T/-308G	Ref	
		-1031C/-308G	**0.67 (0.46–0.97)**	**0.035**
		-1031T/-308A	0.99 (0.62–1.56)	0.954
		-1031C/-308A	-	-
-1031T>C	-238G>A	-1031T/-238G	Ref	
		-1031C/-238G	0.75 (0.50–1.11)	0.158
		-1031C/-238A	-	-
		-1031T/-238A	-	-
-857C>T	-376G>A	-857C/-376G	Ref	
		-857C/-376A	0.49 (0.14–1.75)	0.273
		-857T/-376G	1.10 (0.78–1.54)	0.581
		-857T/-376A	-	-
-857C>T	-308G>A	-857C/-308G	Ref	
		-857T/-308G	1.13 (0.80–1.60)	0.479
		-857C/-308A	1.12 (0.71–1.78)	0.611
		-857T/-308A	-	-
-857C>T	-238G>A	-857C/-238G	Ref	
		-857T/-238G	1.09 (0.78–1.53)	0.599
		-857C/-238A	0.74 (0.38–1.43)	0.367
		-857T/-238A	-	-
-308G>A	-376G>A	-308G/-376G	Ref	
		-308A/-376G	1.07 (0.68–1.68)	0.751
		-308G/-376A	0.47 (0.13–1.69)	0.249
		-308A/-376A	-	-
-308G>A	-238G>A	-308G/-238G	Ref	
		-308A/-238G	1.03 (0.65–1.63)	0.895
		-308G/-238A	0.60 (0.30–1.22)	0.159
		-308A/-238A	-	-
-238G>A	-376G>A	-238G/-376G	Ref	
		-238A/-376G	0.84 (0.37–1.86)	0.662
		-238A/-376A	-	-
		-238G/-376A	-	-

Significant p values are reported in bold face.

The haplotype resulting from the combination of the rare *TNFA* -1031 C allele with the common *TNFA* -308 G allele was significantly associated with SpA with an OR lower than 1.00, suggesting its protective role. When SpA were considered singly, the *TNFA* -1031C/-308G haplotype was confirmed to be associated with AS (OR: 0.35, CI: 0.19–0.65; p<0.001), but not with PsA (OR: 0.95, CI: 0.62–1.44; p = 0.793). Among *HLA-B27* positive patients, the *TNFA* -1031C/-308G haplotype was confirmed to exert a protective role for AS (OR: 0.21, CI: 0.09–0.47; p<0.001), while this was not the case of *HLA-B27* negative patients (OR: 0.71, CI: 0.21–2.42; p = 0.585).

By selecting the two SNPs -1031T>C and -308G>A of the *TNFA* gene, the six following genotypes resulting from haplotypes combinations, were inferred: TG/TG, TG/TA, TG/CG, TA/TA, CG/TA, CG/CG. Since the most frequent *TNFA* haplotype was -1031T/-308G while the protective *TNFA* haplotype was -1031C/-308G, genotypes were grouped as follows: common homozygous (TG/TG), carriers of the protective haplotype (CG/TG, CG/TA and CG/CG), and others (TG/TA and TA/TA). Subjects carrying the protective TNFA -1031C/-308G haplotype were less frequently found among AS (10%, χ^2^ = 12.674 and p = 0.002), not among PsA (26%, χ^2^ = 0.213 and p = 0.899), with respect to controls (49%).

**Fig 1:**
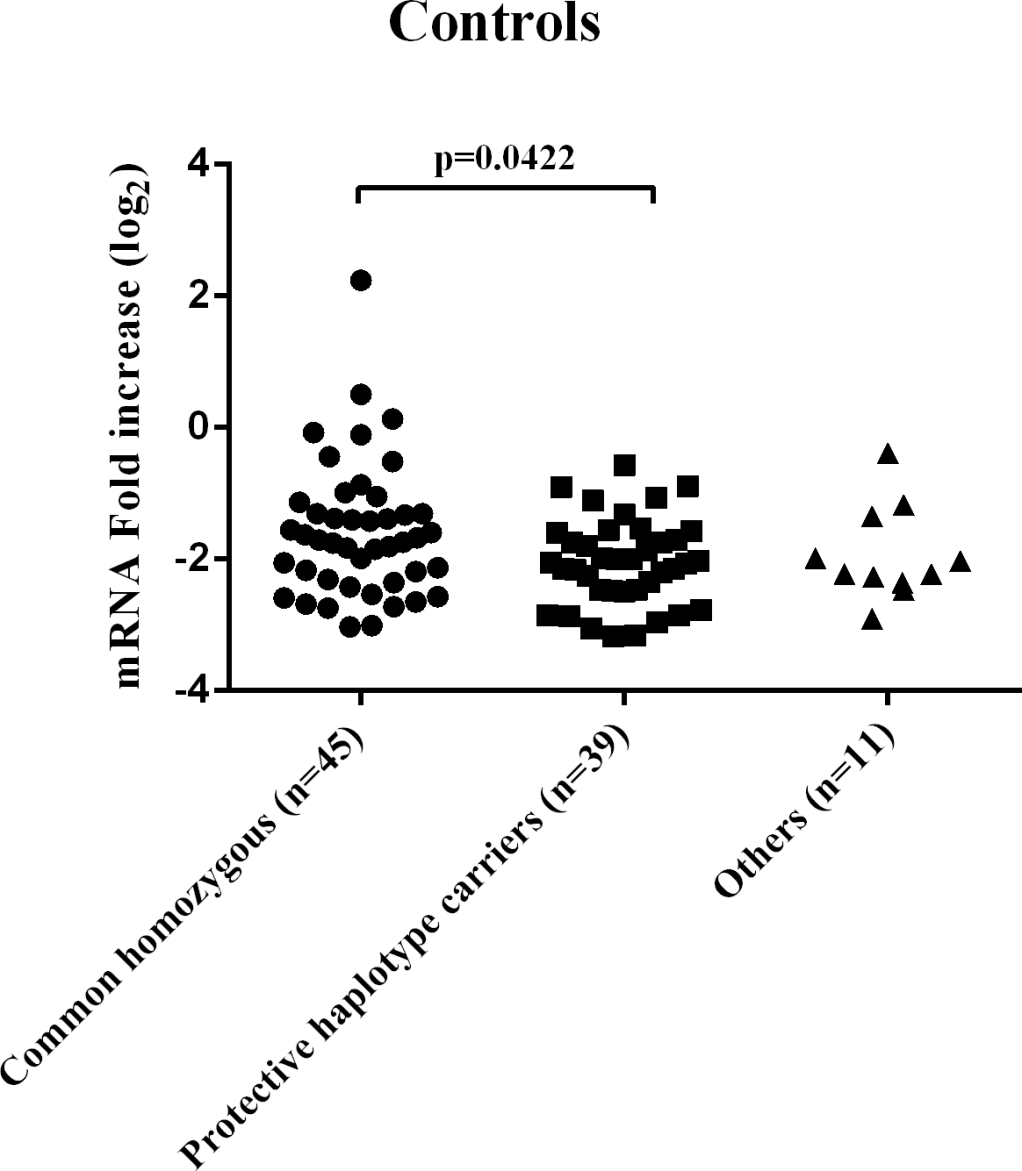
TNF- α mRNA expression in PBMCs obtained from 95 controls grouped on the basis of genotypes derived from the TNFA -1031/-308 haplotypes combinations.

TNF- α expression in PBMCs obtained from a series of 95 controls was found to be significantly reduced among carriers of the protective *TNFA* -1031C/-308G haplotype (Fig 1; F = 3.276, p = 0.042). This association between *TNFA* genetics and TNF- αexpression was not confirmed in SpA patients F = 0.81, p = 0.452).

### *TNFRSF1A* and *MEFV* gene variants do not associate with SpA

To ascertain whether other genes were involved in TNF-α signalling or in joint inflammation, we selected *TNFRSF1A*, which encodes the TNFR-1 of TNF- α, and *MEFV*, which encodes the inflammatory protein pyrin. *TNFRSF1A* and *MEFV* may affect protein function by the presence of uncommon variants. For this reason with the aim to identify any potentially relevant sequence variation, the complete sequence analyses of exons described to carry hotspot mutations more frequently, namely exons 2, 3, 4 and 6 of *TNFRSF1A* gene and exons 2, 3, 5 and 10 of *MEFV* gene, were performed. The sequence analysis of *TNFRSF1A* and *MEFV* genes were performed in an exploratory cohort of 27 controls (25 males, 2 females; mean age ± SD: 44.7 ± 11.9 years) and in 91 cases (57 males, 34 females; mean age ± SD: 52.2 ± 12.5 years). Direct sequencing of exons 2, 3, 4 and 6 of *TNFRSF1A* gene allowed the identification of a total of two SNPs, the R92Q (rs4149584, exon 4) and the c.625+10 A>G (rs1800693, intron 6). None of these two polymorphisms was correlated with SpA (χ^2^ = 1.073 and p = 0.300 for R92Q; χ^2^ = 4.721 and p = 0.094 for c.625+10A>G) (S3 Table).

Direct sequencing of exons 2, 3, 5 and 10 of *MEFV* gene allowed the identification of a total of twenty-one SNPs. Among them, eleven were synonymous SNPs (exon 2: D102D, A165A, G138G; exon 3: R314R; exon 5: E474E, D510D, Q476Q, R501R, Q489Q; exon 10: F721F, P706P), and ten were missense SNPs (exon 2: E148Q, S179N, R202Q; exon 3: P369S, R408Q, R348H; exon 5: A457V; exon 10: A744S; K695M, M680I) of potential functional significance.

Among the ten identified missense SNPs, nine were extremely rare in our population, being their MAF lower or equal to 0.025. Only the R202Q SNP (rs224222) had a MAF of 0.288 and the rare homozygous AA genotype was found only among cases (AS frequency = 0.09; PsA frequency = 0.10), not in controls (χ^2^ = 7.4837; p = 0.006) (S4 Table). To improve the estimate of this association, the number of examined controls was extended from 27 to 218 reaching a ratio between cases and controls close to 1:2. By doing this, the association between the R202Q SNP and SpA was not confirmed (χ^2^ = 1.05, p = 0.59) (S5 Table).

### *TNFRSF1A* c.625+10A>G SNP predicts response to anti-TNFα treatment

Therapy with TNF- α inhibitors although effective, may fail in a subset of SpA patients. Based on the hypothesis that a different genetics background underlying the TNF- α pathway may condition the clinical response, we verified whether *HLA-B27*, *TNFA* and *TNFRSF1A* genetics correlate with response to TNF-α inhibitors. The analysis was performed considering a subset of 65 patients who were under treatment with TNF-α inhibitors and for whom the aforementioned genetic data were available. Thirty six patients had early remission, i.e. BASDAI score lower than/equal to 4 in a time ranging from 1 to 10 months (mean ± SD: 5.94 ± 1.19 months; median: 6 months), while 29 patients were late responders, i.e BASDAI score lower than/equal to 4 in a time ranging from 10 to 36 months (mean ± SD: 16.55 ± 7.16 months; median: 12 months).

Only the *TNFRSF1A* c.625+10A>G SNP was associated with response to therapy, the rare GG genotype being more frequently found among late than early responders (Table 6), the association being confirmed among patients treated with infliximab (Fisher’s exact = 0.026) but not in those treated with adalimumab (Fisher’s exact = 1.000). The number of patients treated with the other TNF- α inhibitors was too low to support statistical analysis and these drugs were therefore not considered in separate analyses.

**Table 6:** *HLA* alleles, *TNFA* and *TNFRSF1A* genetics and response to TNF-α inhibitors.

	BASDAI ≤ 4		
	Early responders	Late responders	
	n (%)	n (%)	
***HLA-B27***			
Negative	22 (33.8%)	20 (30.8%)	χ^2^ = 0.4334
Positive	14 (21.5%)	9 (13.8%)	p = 0.510
***TNFA***			
**-1031T>C**			
T/T	23 (35.4%)	17 (26.1%)	χ^2^ = 0.1960
T/C	11 (16.9%)	10 (15.4%)	p = 0.907
C/C	2 (3.1%)	2 (3.1%)	
**-857C>T**			
C/C	22 (34.9%)	16 (25.4%)	χ^2^ = 4.3876
T/C	14 (22.2%)	8 (12.7%)	p = 0.111
T/T	0 (0.0%)	3 (4.8%)	
**-376G>A**			
G/G	34 (55.7%)	26 (42.6%)	χ^2^ = 0.7552
G/A	1 (1.6%)	0 (0.0%)	p = 0.385
A/A	-	-	
**-308G>A**			
G/G	30 (47.6%)	20 (31.7%)	χ^2^ = 0.8077
G/A	6 (9.5%)	7 (11.1%)	p = 0.369
A/A	-	-	
**-238G>A**			
G/G	33 (50.8%)	27 (41.5%)	χ^2^ = 0.0467
G/A	3 (4.6%)	2 (3.1%)	p = 0.829
A/A	-	-	
***TNFRSF1A***			
**R92Q**			
G/G	35 (53.8%)	26 (40%)	χ^2^ = 1.5925
G/A	1 (1.5%)	3 (4.6%)	p = 0.207
A/A	-	-	
**c.625+10A>G**			
A/A	13 (20%)	5 (7.7%)	χ^2^ = 6.9648
G/A	18 (27.7%)	12 (18.5%)	p = **0.031**
G/G	5 (7.7%)	12 (18.5%)	

Significant p values are reported in bold face.

## Discussion

The SpA are a group of chronic inflammatory diseases characterized by the sharing of distinctive pathological, clinical and radiographic aspects and a strong genetic predisposition [1]. Since no specific biomarkers of diagnosis and prognosis have been validated for the clinical assessment of these diseases [5,6], we verified whether inflammatory biochemical markers commonly used in clinical practice and genetic variants of *HLA* locus, *TNF-TNFR* pathway and *MEFV* play a part in increasing the risk of the most prevalent SpA subtypes, AS or PsA, and/or in predicting the response to therapy with TNF-α inhibitors.

The two SpA subtypes, as expected, were well distinguished by characteristic clinical manifestations. Family history of disease, personal history of psoriasis, dactylitis and peripheral arthritis were more frequently found among PsA, and this fit well with the CASPAR criteria [26]. Back pain and buttock pain, although commonly shared by the two disease groups, were more frequent among AS than PsA, while some extra-articular clinical manifestations were extremely rare in both groups.

In both AS and PsA, WBC, PMN, PLT and serum CRP were higher than in controls, although often comprised within the reference intervals. These minimal differences in systemic signs of inflammation between cases and controls might depend on treatment (all patients were on TNF-α inhibitors or nonsteroidal anti-inflammatory drugs-NSAIDs/disease-modifying antirheumatic drugs- DMARDS) and indicate that the systemic inflammatory process, underlying these diseases, cannot be completely switch off [32].

The main genetic determinant of SpA, *HLA-B27*, was confirmed in the present study to be strictly associated with AS, and less strictly with PsA [33]. Located in proximity of the *HLA* genes, the *TNFA* gene appears of potential interest in SpA. Five main polymorphisms in the promoter region of this gene have been described in Caucasians and reported to be associated with different inflammatory and autoimmune diseases [34,35]. Considering the specific SpA setting, data in the literature are not definitive and sometimes contrastive, not only because of differences in ethnicity, number and selection criteria of the studied populations, but also because these SNPs regulate *TNFA* gene transcription mainly acting as haplotypes, which were verified in this study. Only *TNFA* -1031T>C SNP was associated with SpA diagnosis, while the other studied SNPs were not, this finding being in agreement with previous data [8]. The haplotypes deriving from the pairwise combinations of the five studied SNPs were inferred and only the haplotype -1031C/-308G was significantly associated with SpA, exerting in these diseases a protective role, which was mainly for AS and confirmed also among *HLA-B27* positive patients. This protective role might be explained taking into account that any single allele concurring in determining the studied haplotype was demonstrated to be associated with reduced TNF- α release. In fact, the rare *-*1031C allele, in complete linkage disequilibrium with the -863A allele, associates with low TNF- α production [36], and the -308G common allele associates with a lower spontaneous or stimulated TNF- α release both *in vitro* and *in vivo* with respect to the rare -308A allele [37,38]. The hypothesis that *TNFA* -1031C/-308G haplotype might determine a reduced TNF- α transcription was confirmed in this study by the analysis of mRNA expression levels in PBMCs obtained from healthy subjects. However, in the presence of diseases, the dampened TNF- α expression associated with *TNFA* -1031C/-308G haplotype was not seen. It is not surprising because of the limited sample size and the complicate situations of patients, such of treatment of TNF- α inhibitors. Based on the above data, it is reasonable to suggest that the *TNFA* -1031C/-308G haplotype exerts a protective role for SpA, but mainly for AS, in *HLA-B27* positive subjects by limiting the transcription of TNF- α.

In the complex scenario of TNF- α effects in inflammation and autoimmunity one might bear in mind the role of TNF- α receptors, which genetic variability might underlies complex autoinflammatory diseases such as TRAPS [17]. Only a few number of variants of *TNFRSF1A*, encoding for the TNFR-1, were detected in our studied population and none of them was associated with the diagnosis of AS or PsA. Therefore, in the absence of copy number variation or other mutations, a role for *TNFRSF1A* genetics in SpA could be reasonably excluded.

Other genes involved in the inflammation processes, as genes encoding cytokines (e.g. *IL23R*, *IL17*, *IL2R*) or proteins of the inflammasome pathway (e.g. *CARD9*, *CARD14*, *CARD16*) have been reported as associated with SpA in several studies [9,39]. An interesting candidate involved in inflammasome pathway is *MEFV* gene, encoding for the protein pyrin, critically involved in the autoinflammatory disease FMF and recently suggested as potentially involved also in SpA [18–24]. Several *MEFV* variants were identified in our studied population, half without functional significance and half with potential functional consequences for the predicted amino acid substitutions in the protein. These variants were however sporadic and none was associated with SpA diagnosis. Intriguingly the main *MEFV* variant that has been demonstrated to be associated with AS, namely the M694V [21,23,24], was never recorded among our series of patients. These apparent discrepant results are probably consequent to differences in ethnicity (Italian vs Turkish) and in patient’s selection criteria (SpA patients in our series vs FMF patients with AS in the Turkish series).

One of the main problems in the clinical setting of SpA is the interindividual variability in response to therapy, mainly in response to TNF-α inhibitors [40–42]. Different drugs belonging to this category are now available and they are frequently used to treat both AS and PsA especially when NSAIDs or DMARDS are not beneficial [43,44]. In this study about 88% AS and 59% PsA were on treatment with TNF-α inhibitors, and among them 11% AS and 19% PsA required in their disease history the switch from one to at least another drug type for an incomplete response to treatment. This empirical approach is the only one actually proposed since no predictive response biomarkers are validated. Their identification is really necessary also considering the high costs for the health care system of this type of therapy [45]. Among factors that might affect variability in response to therapy, genetics should be considered besides physiological (age, sex, weight, and body fat), pathophysiological (liver, kidney, and cardiovascular function, and associated diseases), and environmental factors (tobacco and alcohol consumption and concomitant treatments) [46–48]. In our series carriers of rare c.625+10 G allele of *TNFRSF1A* gene were mainly late responders. This polymorphism might affect the response to TNF-α inhibitors because it is associated with an altered TNF-α/TNFR1 balance. In fact, patients carrying the rare G allele have been suggested to synthesize a truncated TNFR-1 [49], and the common A allele was previously demonstrated by us to be correlated with increased serum TNF-α levels in patients with celiac disease [30].

Although a limitation of this study is represented by the lack of complete genetic data for all enrolled patients and controls, any group of data were obtained by a significant number of patients and controls. Future perspectives are confirmatory studies on genetics as predictor of the response to TNF- α inhibitors. In this setting, besides *TNFRSF1A*, many other genes of the TNF/TNFR pathway and of related pathways should be considered and comprehensively investigated possibly through new technologies, like next generation sequencing, in large series of patients to support clinical translation of results.

In conclusion the results of this study indicate the relevant role of TNF-TNFR pathway genetics in the complex network causing SpA and conditioning response to therapy. *TNFA* was shown to be a predisposing factor for SpA, but mainly for AS, among Italian patients, while genetics of the autoinflammatory gene *MEFV* appears of no impact in this setting. The haplotype resulting from *TNFA*-1031C/-308G, associated with lower TNF- α production, exerts a protective role in AS, while the *TNFRSF1A* c.625+10A>G polymorphism emerged as a potential predictor of response to TNF-α inhibitors.

## Supporting information

S1 Fig Genotype discrimination of the *TNFA* -857 C>T SNP. Images show the representative TaqMan amplification curves of samples homozygous C/C, heterozygous C/T and homozygous T/T obtained using 857 C (upper) and T (lower) probes separately. Allelic discrimination scatter plot for the *TNFA* -857 C>T is shown on the right.
(TIF)

S2 Fig Allelic discrimination scatter plots. Allelic discrimination scatter plots for the *TNFA* gene SNPs: -1031T>C and -376G>A (upper panel), -308G>A and -238G>A (lower panel).
(TIF)

S1 File Materials and methods. *MEFV* gene analysis.
(DOC)

S1 Table Primer sequences for the amplification of exons 2, 3, 5 and 10 of the *MEFV* gene. (DOC)

S2 Table TNFA haplotypes combinations: Frequencies inferred from controls. The Table reports the haplotypes combinations (and their respective frequencies) statistically inferred from the pairwise combination of *TNFA*-1031T>C, -857C>T, -376G>A, -308G>A and -238G>A polymorphisms in control subjects.
(DOC)

S3 Table *TNFRSF1A* gene polymorphisms in controls and cases (AS and PsA). Results from the exploratory study. Table reports the genotypes and Minor Allele Frequency (MAF) resulting from the two SNPs, identified in patients (AS and PsA) and controls (explanatory group), through the DHPLC screening of exons 2, 3, 4 and 6 of the *TNFRSF1A* gene followed by direct sequence analysis of positive samples.
(DOC)

S4 Table *MEFV* gene missense polymorphisms in controls and in cases (AS and PsA patients). Results from the exploratory study. Table reports the genotypes and Minor Allele Frequency (MAF) resulting from the ten missense polymorphisms, identified in patients (AS and PsA) and controls (explanatory group), through the direct sequencing of exons 2, 3, 5 and 10 of *MEFV* gene.
(DOC)

S5 Table R202Q polymorphism in the entire studied population. Table reports the genotypes and Minor Allele Frequency (MAF) resulting from the genotype discrimination of the R202Q SNP (rs224222) in *MEFV* gene (exon 2), in the entire studied population.
(DOC)

## Abbreviations

ALT: Alanine aminotransferase
ANOVA: Analysis of variance
AS: Ankylosing Spondylitis
ASDAS: Ankylosing Spondylitis Disease Activity Score
BASDAI: Bath Ankylosing Spondylitis Disease Activity Index
BASFI: Bath Ankylosing Spondylitis Functional Index
BASMI: Bath Ankylosing Spondylitis Metrology Index
BMI: Body Mass Index
CASPAR: ClASsification criteria for Psoriatic Arthritis
CI: 95% confidence intervals
CRP: C-reactive protein
DAS: Disease Activity Score
DHPLC: denaturing high-performance liquid chromatography
DMARDS: disease-modifying antirheumatic drugs
ESR: erythrocyte sedimentation rate
EULAR: European League Against Rheumatism
FMF: Familial Mediterranean Fever
HAQ: Health Assessment Questionnaire
HPRT1: Hypoxanthine-guanine phosphoribosyltransferase
HLA-B27: Human Leukocyte Antigen B27
IBD: Inflammatory Bowel Diseases
MHC: major histocompatibility complex
MMP3: matrix metalloproteinase-3
MEFV: Mediterranean fever
MAF: Minor Allele Frequency
NSAIDs: nonsteroidal anti-inflammatory drugs
OR: Odds ratio
PBMCs: Peripheral blood mononuclear cells
PLT: platelets
PMN: polymorphonuclear cells
PsA: Psoriatic Arthritis
SD: Standard Deviation
SNPs: Single nucleotide polymorphisms
SpA: Spondyloarthritis
TNF-α: tumor necrosis factor alpha
TNFR: TNF receptor
TNFRSF1A: TNF receptor superfamily member 1A
TNFSF15: TNF ligand superfamily, member 15
TRADD: TNF receptor type 1-associated death domain protein
TRAPS: tumor necrosis factor receptors associated syndrome
VEGF: vascular endothelial growth factor
WBC: white blood cells
