# Supplementary figures and images for: Genetics in TNF-TNFR pathway: A complex network causing spondyloarthritis and conditioning response to anti-TNFα therapy
Source: PLoS One. 2018 Mar 26;13(3):e0194693. doi: 10.1371/journal.pone.0194693 (PMC5868803; doi:10.1371/journal.pone.0194693)

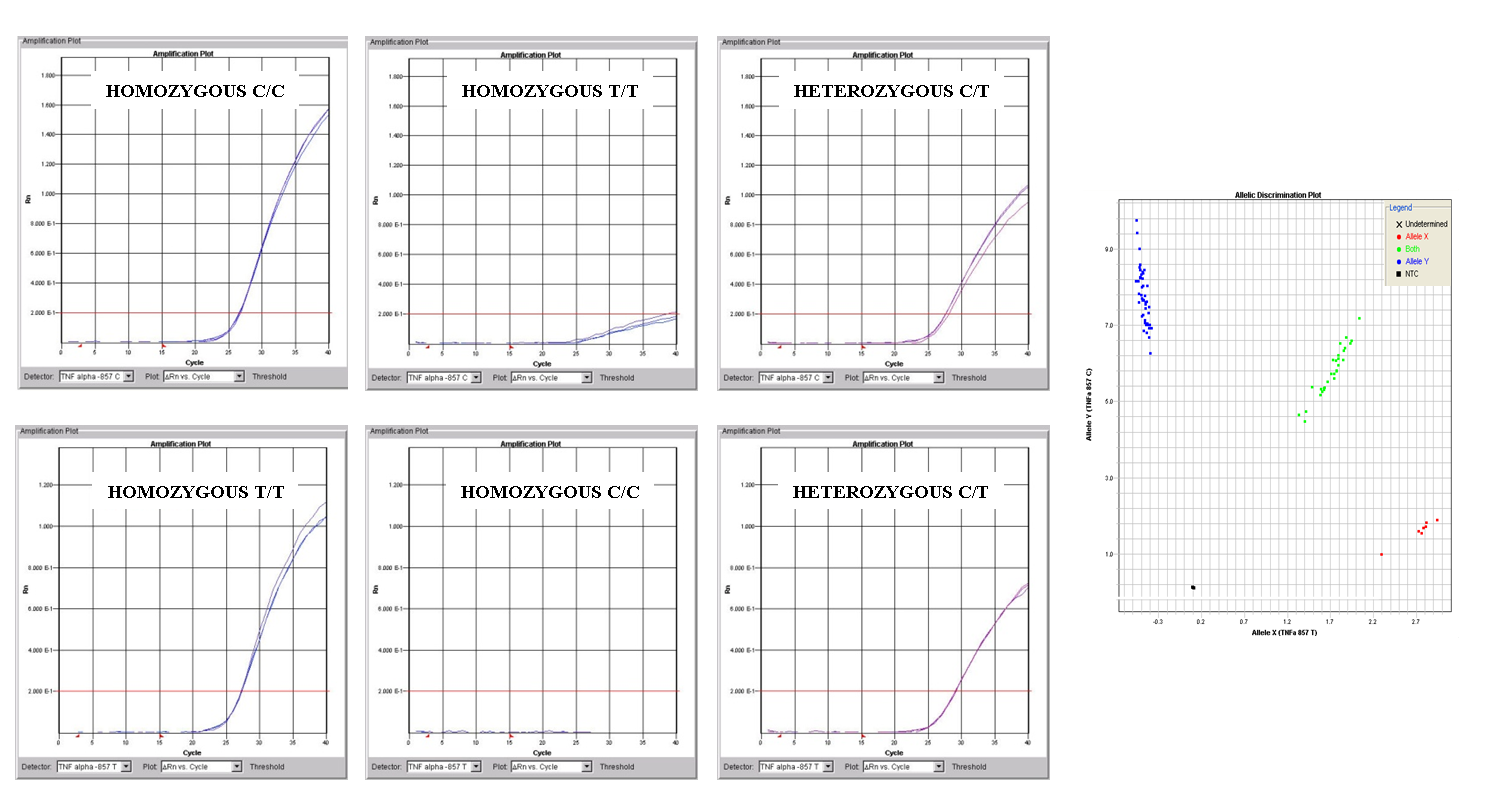

Supplement: S1 Fig — Images show the representative TaqMan amplification curves of samples homozygous C/C, heterozygous C/T and homozygous T/T obtained using 857 C (upper) and T (lower) probes separately. Allelic discrimination scatter plot for the TNFA -857 C>T is shown on the right. (TIF) [file pone.0194693.s001.tif]

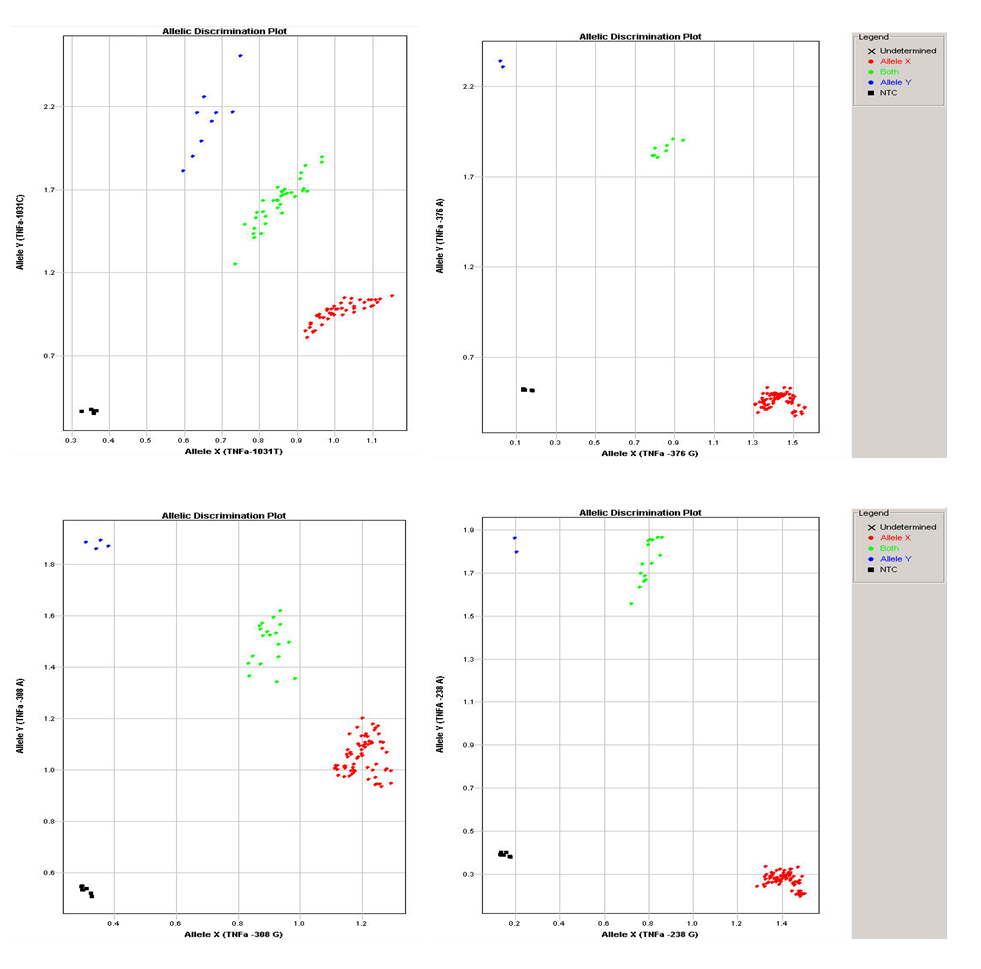

Supplement: S2 Fig — Allelic discrimination scatter plots for the TNFA gene SNPs: -1031T>C and -376G>A (upper panel), -308G>A and -238G>A (lower panel). (TIF) [file pone.0194693.s002.tif]
