# Supplementary material for: Genetics in TNF-TNFR pathway: A complex network causing spondyloarthritis and conditioning response to anti-TNFα therapy
Source: PLoS One. 2018 Mar 26;13(3):e0194693. doi: 10.1371/journal.pone.0194693 (PMC5868803; doi:10.1371/journal.pone.0194693)
Supplement: S1 File — MEFV gene analysis. (DOC) [file pone.0194693.s003.doc]

**S1 File. Materials and Methods**

***MEFV* gene analysis**

For each exon, 100 ng of genomic DNA were amplified in a reaction mix composed by 500 nM of each forward and reverse primer (Supplementary Table 1), 200 µM of each deoxynucleotide triphosphates (dNTPs), 2.5 mM of MgCl_2_, 1 Unit (U) of Taq polymerase (Super Taq, AB Analitica, Padova, Italy), 1X manufacturer-provided buffer, 10% dimethyl sulfoxide (DMSO) (only for exon 2) in a final volume of 25 µl. PCR reactions were run with an initial denaturation at 94 °C for 5 minutes, followed by a touchdown step (94 °C for 30 seconds, 68°C in the first cycle for 30 seconds and -0.5 °C/cycle for 9 cycles, 72°C for 45 seconds) and more 26 cycles at 94 °C for 30 seconds, 62 °C for 30 seconds, 72 °C for 45 seconds, with a final extension at 72 °C for 7 minutes. Water was used as negative control in each PCR run. After amplification of DNA, to verify the presence of amplification products and to exclude the presence of contamination in mixes, gel electrophoresis of 10 μl of the amplification products was performed using 2% agarose gels (E-Gel, Invitrogen, Life Technologies, Monza, Italy) with ethidium bromide as staining.

MEFV gene sequencing was performed on the automatic sequencer 3130ABI PRISM Genetic Analyzer (Applied Biosystem, CA, USA), using 2 µL of previously purified (ExoSAP, GE Healthcare, Fairfield, CT, USA) amplification product mixed with 300 nM of each forward and reverse PCR primer, 4 μl of Big Dye terminator Mix (Applied Biosystem, CA, USA) and 5% DMSO (only for exon 2) in a final volume of 20 µL. Two additional internal primers were also used to perform the sequence of the exon 2 (Fint: 5’-CCCTGAGCAAACGCAGAG-3’ and Rint: 5’- GTACACTTCGAAGGGCCTGC-3’). Chromatograms were analysed with Chromas Lite 2.6.1 software (Technelysium Pty Ltd., South Brisbane, QLD, Australia).
