# Supplementary material for: Genetics in TNF-TNFR pathway: A complex network causing spondyloarthritis and conditioning response to anti-TNFα therapy
Source: PLoS One. 2018 Mar 26;13(3):e0194693. doi: 10.1371/journal.pone.0194693 (PMC5868803; doi:10.1371/journal.pone.0194693)
Supplement: S1 Table — (DOC) [file pone.0194693.s004.doc]

**S1 Table**. Primer sequences for the amplification of exons 2, 3, 5 and 10 of the *MEFV* gene.

| **Exon** | **Forward and reverse primer sequence** | **Amplicon (bp)** |
| --- | --- | --- |
| 2 | F-5’-GGGGA TTCTCTCTCCTCTGC-3’ | 903 |
|  | R-5’-CTCAAAGTCTTGGCCTCCAG-3’ |  |
| 3 | F-5’-AACTTGGGTTTGCCATTCAG-3’ | 438 |
|  | R-5’-AAGTGCCTGGCAGAGAAGAG-3’ |  |
| 5 | F-5’-CCACCTCTTATCCACCTCCA-3’ | 449 |
|  | R-5’-CTAGGCCTTAGGGGCTTCAC-3’ |  |
| 10 | F-5’-CAGGTGGGGAGAACCCTGTAG-3’ | 757 |
|  | R-5’-CCGTGGGCACAGTAACTATT-3’ |  |

F: forward primer, R: reverse primer, bp: base pair.
