# Supplementary material for: Genetics in TNF-TNFR pathway: A complex network causing spondyloarthritis and conditioning response to anti-TNFα therapy
Source: PLoS One. 2018 Mar 26;13(3):e0194693. doi: 10.1371/journal.pone.0194693 (PMC5868803; doi:10.1371/journal.pone.0194693)
Supplement: S2 Table — The Table reports the haplotypes combinations (and their respective frequencies) statistically inferred from the pairwise combination of TNFA-1031T>C, -857C>T, -376G>A, -308G>A and -238G>A polymorphisms in control subjects. (DOC) [file pone.0194693.s005.doc]

**S2 Table.** *TNFA* haplotypes combinations: frequencies inferred from controls.

| **SNPs** | **SNPs** | **TNFA haplotypes** | **Frequencies (%)** |
| --- | --- | --- | --- |
| -1031T>C | -857C>T | -1031T/-857C | 51.87 |
|  |  | -1031C/-857C | 27.05 |
|  |  | -1031T/-857T | 21.07 |
|  |  | -1031C/-857T | <0.4 |
| -1031T>C | -376G>A | -1031T/-376G | 72.83 |
|  |  | -1031C/-376G | 24.79 |
|  |  | -1031C/-376A | 2.37 |
|  |  | -1031T/-376A | <0.4 |
| -1031T>C | -308G>A | -1031T/-308G | 62.38 |
|  |  | -1031C/-308G | 27.35 |
|  |  | -1031T/-308A | 10.28 |
|  |  | -1031C/-308A | <0.4 |
| -1031T>C | -238G>A | -1031T/-238G | 73.34 |
|  |  | -1031C/-238G | 20.66 |
|  |  | -1031C/-238A | 6.00 |
|  |  | -1031T/-238A | <0.4 |
| -857C>T | -376G>A | -857C/-376G | 76.54 |
|  |  | -857T/-376G | 21.07 |
|  |  | -857C/-376A | 2.38 |
|  |  | -857T/-376A | <0.4 |
| -857C>T | -308G>A | -857C/-308G | 68.66 |
|  |  | -857T/-308G | 21.07 |
|  |  | -857C/-308A | 10.26 |
|  |  | -857T/-308A | <0.4 |
| -857C>T | -238G>A | -857C/-238G | 72.95 |
|  |  | -857T/-238G | 21.07 |
|  |  | -857C/-238A | 5.97 |
|  |  | -857T/-238A | <0.4 |
| -308G>A | -376G>A | -308G/-376G | 87.29 |
|  |  | -308A/-376G | 10.28 |
|  |  | -308G/-376A | 2.43 |
|  |  | -308A/-376A | <0.4 |
| -308G>A | -238G>A | -308G/-238G | 83.74 |
|  |  | -308A/-238G | 10.28 |
|  |  | -308G/-238A | 5.98 |
|  |  | -308A/-238A | <0.4 |
| -238G>A | -376G>A | -238G/-376G | 94.04 |
|  |  | -238A/-376G | 3.67 |
|  |  | -238A/-376A | 2.28 |
|  |  | -238G/-376A | <0.4 |
