# Supplementary material for: Genetics in TNF-TNFR pathway: A complex network causing spondyloarthritis and conditioning response to anti-TNFα therapy
Source: PLoS One. 2018 Mar 26;13(3):e0194693. doi: 10.1371/journal.pone.0194693 (PMC5868803; doi:10.1371/journal.pone.0194693)
Supplement: S3 Table — Table reports the genotypes and Minor Allele Frequency (MAF) resulting from the two SNPs, identified in patients (AS and PsA) and controls (explanatory group), through the DHPLC screening of exons 2, 3, 4 and 6 of the TNFRSF1A gene followed by direct sequence analysis of positive samples. (DOC) [file pone.0194693.s006.doc]

**S3 Table.** *TNFRSF1A* gene polymorphisms in controls and patients (AS and PsA).

**Results from the exploratory study.**

| **dbSNP**  ***TNFRSF1A* gene** | **MAF** | **Controls (n=27)** | | | **AS (n=36)** | | | **PsA (n=55)** | | | **χ^2^, p** |
| --- | --- | --- | --- | --- | --- | --- | --- | --- | --- | --- | --- |
|  |  | **GENOTYPES** | | | **GENOTYPES** | | | **GENOTYPES** | | |  |
|  |  | **Number** | | | **Number** | | | **Number** | | |  |
|  |  | **(frequency)** | | | **(frequency)** | | | **(frequency)** | | |  |
| R92Q | A  (0.034) | A/A | G/A | G/G | A/A | G/A | G/G | A/A | G/A | G/G | χ^2^= 1.04  p= 0.60 |
| c.362G>A |  | 0 | 3 | 24 | 0 | 2 | 34 | 0 | 3 | 52 |  |
| rs4149584 |  | (-) | (0.11) | (0.89) | (-) | (0.05) | (0.95) | (-) | (0.05) | (0.95) |  |
| c.625+10A>G rs1800693 | G  (0.483) | G/G | G/A | A/A | G/G | G/A | A/A | G/G | G/A | A/A | χ^2^= 5.22  p= 0.27 |
|  |  | 4 | 19 | 4 | 8 | 16 | 12 | 14 | 27 | 14 |  |
|  |  | (0.015) | (0.70) | (0.15) | (0.22) | (0.45) | (0.33) | (0.25) | (0.50) | (0.25) |  |
