# Supplementary material for: Genetics in TNF-TNFR pathway: A complex network causing spondyloarthritis and conditioning response to anti-TNFα therapy
Source: PLoS One. 2018 Mar 26;13(3):e0194693. doi: 10.1371/journal.pone.0194693 (PMC5868803; doi:10.1371/journal.pone.0194693)
Supplement: S4 Table — Table reports the genotypes and Minor Allele Frequency (MAF) resulting from the ten missense polymorphisms, identified in patients (AS and PsA) and controls (explanatory group), through the direct sequencing of exons 2, 3, 5 and 10 of MEFV gene. (DOC) [file pone.0194693.s007.doc]

**S4 Table.** *MEFV* gene missense polymorphisms in controls and in cases (AS and PsA patients).

Results from the exploratory study.

| **dbSNP**  ***MEFV* gene** | **MAF** | **Cases (n=91)** | | | **Controls (n=27)** | | | **χ^2^, p** |
| --- | --- | --- | --- | --- | --- | --- | --- | --- |
|  |  | **GENOTYPES** | | | **GENOTYPES** | | |  |
|  |  | **Number** | | | **Number** | | |  |
|  |  | **(frequency)** | | | **(frequency)** | | |  |
| R202Q | A  (0.288) | A/A | G/A | G/G | A/A | G/A | G/G | χ2=7.48  **p=0.006** |
| c.605G>A |  | 8 | 39 | 44 | 0 | 13 | 14 |  |
| rs224222 |  | (0.09) | (0.43) | (0.48) | (-) | (0.48) | (0.52) |  |
| S179N  c.536G>A | A  (0.004) | A/A | G/A | G/G | A/A | G/A | G/G | χ2= 0.30  p= 0.59 |
|  |  | 0 | 1 | 90 | 0 | 0 | 27 |  |
|  |  | (-) | (0.01) | (0.99) | (-) | (-) | (1.00) |  |
| E148Q | C  (0.013) | C/C | G/C | G/G | C/C | G/A | G/G | χ2= 0.90  p= 0.34 |
| c.442G>C |  | 0 | 3 | 88 | 0 | 0 | 27 |  |
| rs3743930 |  | (-) | (0.03) | (0.97) | (-) | (-) | (1.00) |  |
| P369S | T  (0.025) | T/T | C/T | C/C | T/T | C/T | C/C | χ2= 3.48  p= 0.18 |
| c.1105C>T |  | 0 | 3 | 88 | 1 | 1 | 25 |  |
| rs11466023 |  | (-) | (0.03) | (0.97) | (0.04) | (0.04) | (0.92) |  |
| R408Q | A  (0.017) | A/A | G/A | G/G | A/A | G/A | G/G | χ2= 0.01  p= 0.91 |
| c.1223G>A |  | 0 | 3 | 88 | 0 | 1 | 26 |  |
| rs11466024 |  | (-) | (0.03) | (0.97) | (-) | (0.04) | (0.96) |  |
| R348H | A  (0.004) | A/A | G/A | G/G | A/A | G/A | G/G | χ2= 0.30  p= 0.59 |
| c.1043G>A |  | 0 | 1 | 90 | 0 | 0 | 27 |  |
| rs104895198 |  | (-) | (0.01) | (0.99) | (-) | (-) | (1.00) |  |
| A457V | T  (0.004) | T/T | C/T | C/C | T/T | C/T | C/C | χ2= 0.30  p= 0.59 |
| c.1370C>T |  | 0 | 1 | 90 | 0 | 0 | 27 |  |
| rs104895151 |  | (-) | (0.01) | (0.99) | (-) | (-) | (1.00) |  |
| A744S | T  (0.004) | T/T | G/T | G/G | T/T | G/T | G/G | χ2= 0.30  p= 0.59 |
| c.2230G>T |  | 0 | 1 | 90 | 0 | 0 | 27 |  |
| rs61732874 |  | (-) | (0.01) | (0.99) | (-) | (-) | (1) |  |
| K695M | T  (0.008) | T/T | A/T | A/A | T/T | A/T | A/A | χ2= 0.87  p= 0.35 |
| c.2084A>T |  | 0 | 1 | 90 | 0 | 1 | 26 |  |
| rs104895129 |  | (-) | (0.01) | (0.99) | (-) | (0.04) | (0.96) |  |
| M680I | A  (0.004) | A/A | G/A | G/G | A/A | G/A | G/G | χ2= 0.30  p= 0.59 |
| c.2040G>A |  | 0 | 1 | 90 | 0 | 0 | 27 |  |
| rs28940580 |  | (-) | (0.01) | (0.99) | (-) | (-) | (1.00) |  |
