# Supplementary material for: Genetics in TNF-TNFR pathway: A complex network causing spondyloarthritis and conditioning response to anti-TNFα therapy
Source: PLoS One. 2018 Mar 26;13(3):e0194693. doi: 10.1371/journal.pone.0194693 (PMC5868803; doi:10.1371/journal.pone.0194693)
Supplement: S5 Table — Table reports the genotypes and Minor Allele Frequency (MAF) resulting from the genotype discrimination of the R202Q SNP (rs224222) in MEFV gene (exon 2), in the entire studied population. (DOC) [file pone.0194693.s008.doc]

**S5 Table.** R202Q polymorphism in the entire studied population.

| **Gene** | **dbSNP** | **MAF** | **Cases (n=91)** | | | **Controls (n=218)** | | | **χ^2^, p** |
| --- | --- | --- | --- | --- | --- | --- | --- | --- | --- |
|  |  |  | **GENOTYPES** | | | **GENOTYPES** | | |  |
|  |  |  | **Number** | | | **Number** | | |  |
|  |  |  | **(frequency)** | | | **(frequency)** | | |  |
| ***MEFV*** | R202Q | A  (0.272) | A/A | G/A | G/G | A/A | G/A | G/G | χ^2^= 1.05  p= 0.59 |
|  | c.605G>A |  | 8 | 39 | 44 | 15 | 83 | 120 |  |
|  | rs224222 |  | (0.09) | (0.43) | (0.48) | (0.07) | (0.38) | (0.55) |  |
